# Epithelioid hemangioendothelioma: A rare mimicker of post-traumatic hematoma

**DOI:** 10.18632/oncoscience.629

**Published:** 2025-10-03

**Authors:** Reshmi Sultana, Tushar M. Parmeshwar, Nynasindhu Akula, Abhimanyu Sharma

**Affiliations:** ^1^Department of General Surgery, All India Institute of Medical Sciences, Bibinagar, Hyderabad, Telangana 508126, India; ^2^Department of Pathology, All India Institute of Medical Sciences, Bibinagar, Hyderabad, Telangana 508126, India

**Keywords:** epitheloid haemangioendothelioma, haematoma, vascular neoplasm, subcutaneous swelling, immunohistochemistry

## Abstract

Vascular tumors of intermediate malignancy occupy a perplexing space in oncology; too aggressive to be dismissed, yet too indolent to follow the predictable trajectory of high-grade sarcomas. Among them, epithelioid hemangioendothelioma (EHE) stands out for its rarity, histological subtlety, and unpredictable clinical course. Frequently, its bland cytology, low mitotic activity, and deceptively benign patterns obscure its malignant potential, leading to diagnostic uncertainty. In the present case, the diagnostic challenge was evident, as the lesion presented clinically as a subcutaneous swelling resembling a hematoma. Histopathological examination demonstrated polygonal endothelial cells with intracytoplasmic vacuoles containing erythrocytes indicative of a vascular origin. Definitive diagnosis was achieved through immunohistochemical confirmation, showing positivity for CD31, CD34, and FLI-1. This report aims to highlight the diagnostic nuances, potential for misinterpretation, and therapeutic dilemmas associated with EHE, thereby reinforcing the need for vigilance in evaluating seemingly “low-risk” atypical subcutaneous swellings.

## INTRODUCTION

Epithelioid haemangioendothelioma (EHE) is a rare malignant vascular tumor arising from the proliferation of neoplastic endothelial cells, exhibiting both vascular and epithelial characteristics [1, 2]. First described by Weiss and Enzinger in 1982, EHE is considered an intermediate grade malignancy; more aggressive than benign hemangiomas but less aggressive than conventional angiosarcomas.

EHE can present in diverse patterns, including a solitary lesion, multifocal involvement confined to a single organ or anatomical compartment, or disseminated disease with systemic metastases. Common primary sites include the extremities, lungs, liver, and bones, though it may also occur in the thyroid, palate, mediastinum, lymph nodes, and peritoneum. The clinical presentation often depends on the organ involved, with symptoms ranging from incidental imaging findings to pain, swelling, or organ dysfunction [3].

Due to its rarity and nonspecific clinical features, EHE is frequently misdiagnosed or detected incidentally. Imaging can suggest a vascular tumor, but histopathological examination (HPE) remains the gold standard for diagnosis. Microscopically, EHE shows epithelioid endothelial cells with intracytoplasmic vacuoles, often forming primitive vascular channels. Immunohistochemistry is critical in confirming diagnosis, with tumor cells typically expressing endothelial markers such as CD31, CD34, ERG, and FLI-1 [4].

## CASE REPORT

A 48-year-old woman presented to our outpatient department with a painless swelling over the right leg, first noticed 6 months earlier. The swelling had an insidious onset and gradually increased in size. There was no associated pain, fever, discharge, or history of preceding trauma.

**Figure 1:**
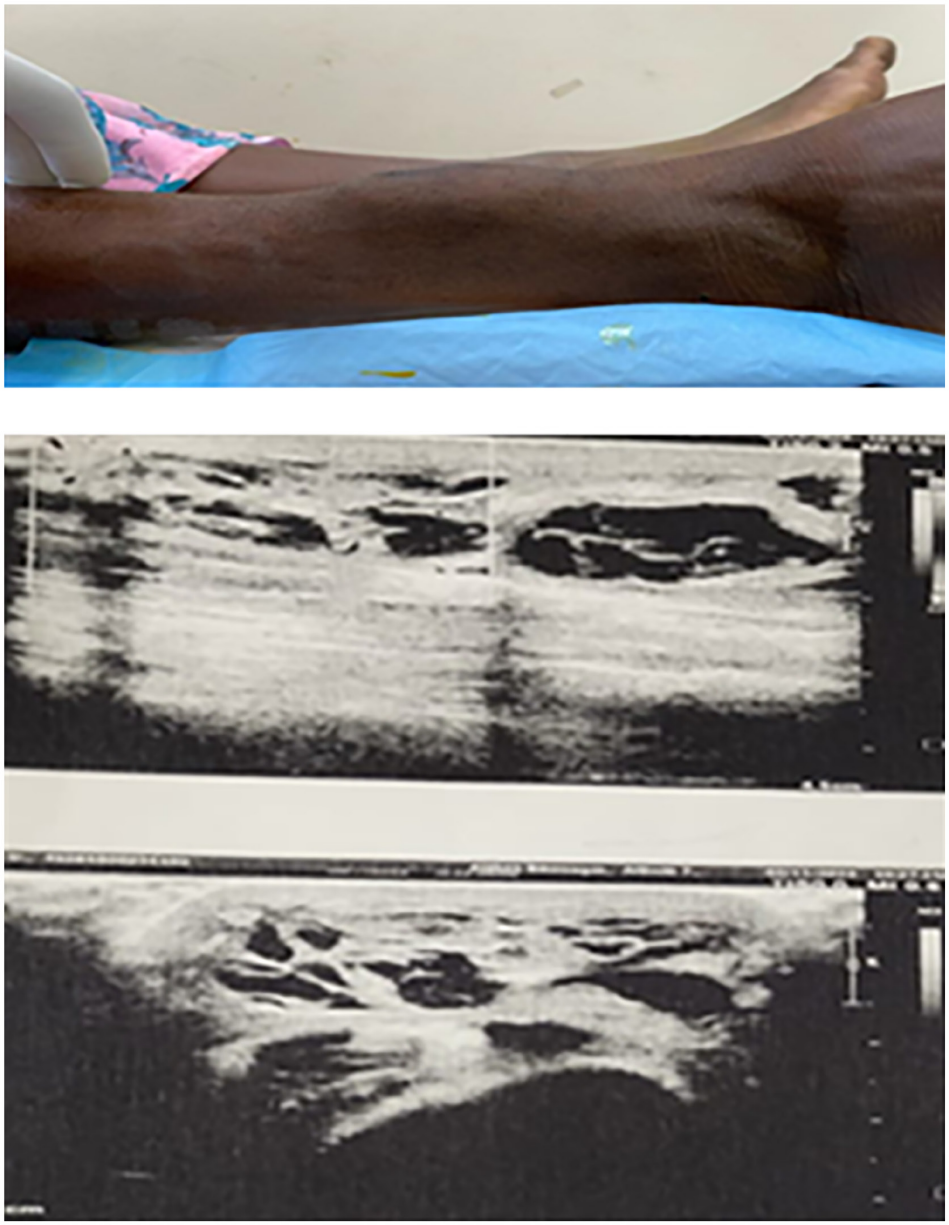
Swelling over anterior aspect of right leg; USG showing hypoechoic cystic lesion measuring 6.8 × 5 cm containing blood as content suggestive of an organised hematoma.

Examination revealed a firm, non-tender, relatively immobile swelling measuring approximately 6 × 5 cm, located on the anterior aspect of the lower third of the right leg. The overlying skin appeared normal, without redness, hyperpigmentation, ulceration, or prominent superficial veins. No regional lymphadenopathy was detected. Routine hematological parameters were within normal limits. Ultrasound of the swelling showed a well-defined, hypoechoic, cystic lesion measuring 6.8 × 5 cm containing internal echoes consistent with blood, suggestive of an organized hematoma (Figure 1). Fine-needle aspiration cytology (FNAC) yielded red blood cells, further supporting this initial impression.

Magnetic resonance imaging (MRI) revealed a well-defined, lobulated, multiloculated lesion in the subcutaneous plane on the anterolateral aspect of the right leg, approximately 6 cm proximal to the lateral malleolus. The lesion was T1-isointense and T2/STIR hyperintense, without diffusion restriction suggesting a benign proliferative lesion.

**Figure 2:**
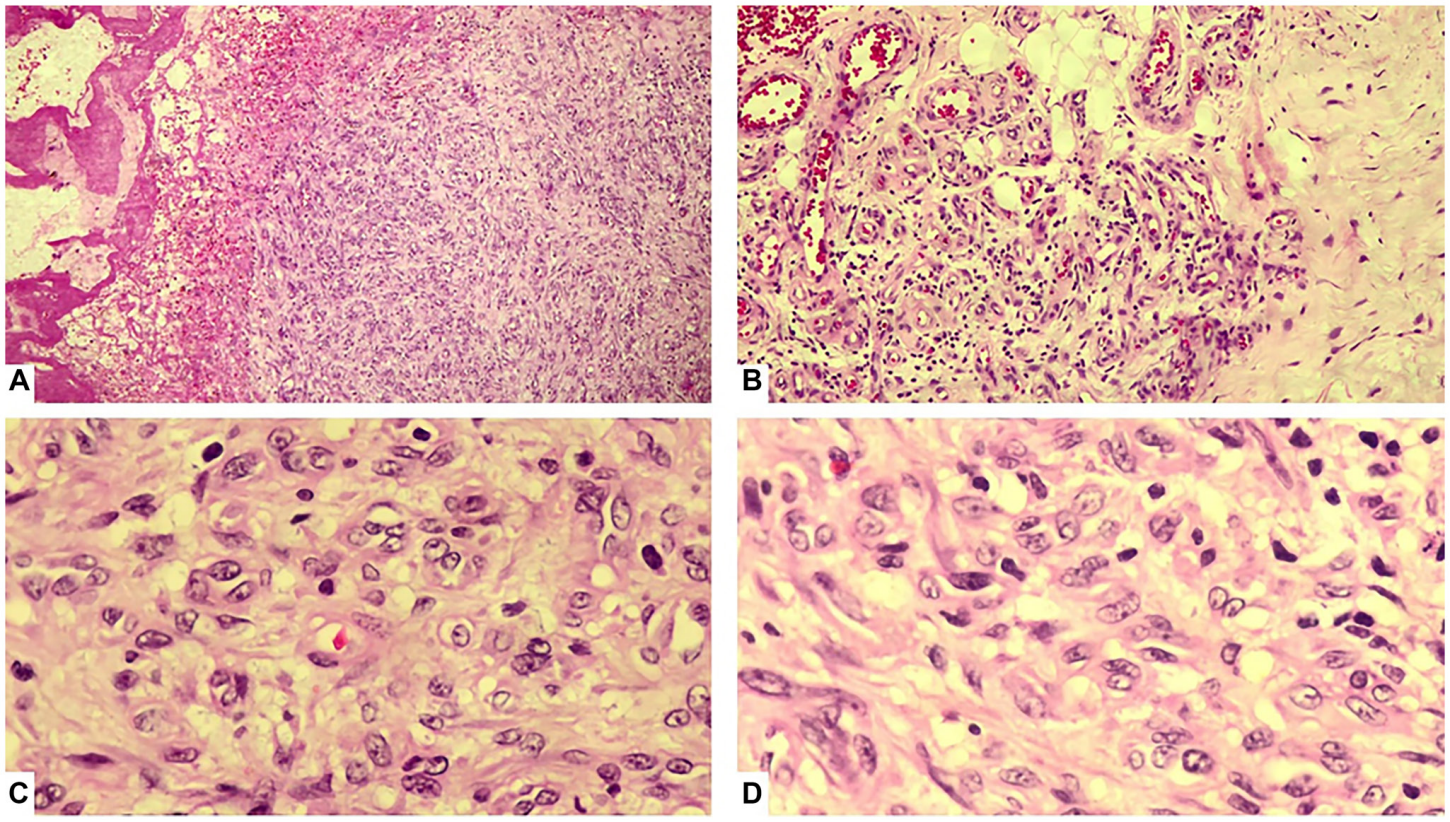
(**A**) Low magnification shows a vasoformative tumour. (**B**) Low magnification showing the tumour infiltrating the surrounding adipose tissue in a myxoid stroma. (**C**) High magnification showing a tumour composed of spindle and epithelioid cells with intracytoplasmic lumina containing erythrocytes. (**D**) High magnification showing a tumour consisting of sheets and nests of spindle and epithelioid cells with intracytoplasmic lumina and minimal atypia.

A wide local excision was performed under local anesthesia. Gross examination showed a well-encapsulated mass. The specimen was submitted for HPE, which gave the diagnosis of EHE with tumor-free surgical margins (Figure 2). The tumor cells exhibited minimal nuclear atypia, vesicular nuclei, and eosinophilic cytoplasm. The mitotic rate was low (<1 mitosis per 10 high-power fields), with no evidence of necrosis. Occasional intracytoplasmic lumina containing erythrocytes were noted. The diagnosis was further confirmed by immunohistochemical staining, which demonstrated strong positivity for endothelial markers CD31, CD34, and factor VIII-related antigen. Fusion gene studies couldn’t be performed due to financial constraints.

Postoperative recovery was uneventful. A metastatic work-up performed in the immediate postoperative period was negative for secondary lesions. At the time of the latest follow-up, there was no evidence of local recurrence or distant metastasis.

## DISCUSSION

EHE is a rare vascular tumor of intermediate malignant potential, most frequently involving the soft tissues of the extremities, lungs, and liver. It belongs to a heterogeneous group of haemangioendotheliomas that also includes retiform, pseudomyogenic, kaposiform, and papillary intralymphatic variants [5]. Although its etiology remains unknown, a predilection for middle-aged women has been observed [6].

### Diagnostic challenges and imaging pitfalls

Soft tissue EHE is diagnostically challenging because its clinical and radiologic presentation often mimics benign entities such as organized hematomas, lipomas, or cystic lesions. USG usually shows discrete hypoechoic lesion and MRI also shows low signal intensity lesions on T1W images and heterogeneous high signal intensity on T2W sequences [7].

In the present case, ultrasonography demonstrated a well-defined hypoechoic lesion containing blood, suggestive of an organized hematoma, while MRI revealed a lobulated, multiloculated lesion with T1 isointensity and heterogeneous T2/STIR hyperintensity. Such overlap is a recognized pitfall in EHE diagnosis, as the tumor’s vascular nature may not produce the aggressive radiologic features typically expected in malignant lesions. This explains why FNAC, which yielded only red blood cells, also failed to identify the neoplastic nature of the lesion.

### Clinical mimics

In the subcutaneous plane, EHE due to its painless, slow-growing nature can resemble post-traumatic hematoma, organized abscess, epidermal inclusion cyst, or benign vascular malformation. A history of trauma, even incidental, may reinforce this impression. Such overlap highlights the need for a broad differential in atypical swellings, especially those with unusual consistency, persistence, or lacking inflammatory signs.

### Rationale for excision

Despite benign-appearing imaging, several factors supported the decision for wide local excision under local anesthesia.

1. The lesion’s progressive enlargement over six months
2. Firm consistency on examination, and
3. Inconclusive FNAC results that did not rule out malignancy.

While a core needle biopsy could have been performed, the lesion’s superficial subcutaneous location, discrete margins, and accessibility made complete excision a reasonable and low-morbidity option, allowing both definitive diagnosis and curative treatment in one step.

### Histopathology and immunohistochemistry

Histologically, epithelioid hemangioendothelioma (EHE) typically shows cords, strands, and nests of epithelioid endothelial cells embedded in a distinctive myxohyaline stroma [8]. The tumor cells are polygonal to slightly spindled, with eosinophilic cytoplasm, vesicular nuclei, and small nucleoli. Intracytoplasmic vacuoles containing erythrocytes, often a subtle indicator of vascular differentiation are present in many cases [9]. Mitotic activity is usually low (<1–2/10 high-power fields), nuclear atypia is generally mild to moderate, and tumor necrosis is uncommon but, when present, may indicate more aggressive behavior. Occasional multinucleated giant cells and stromal sclerosis may also be seen [10].

Immunohistochemically, EHE demonstrates diffuse positivity for vascular markers such as CD31, CD34, ERG, and factor VIII-related antigen, with CD31 being the most sensitive and specific endothelial marker. The tumor is typically negative for epithelial markers (cytokeratin AE1/AE3, EMA) and S100, aiding in the exclusion of carcinoma, melanoma, and most epithelioid sarcomas [11]. CAMTA1 immunoreactivity can also serve as a surrogate for the presence of the WWTR1–CAMTA1 fusion gene [12].

### Molecular genetics

Approximately 90% of EHE cases harbor the t(1;3) (p36;q25) translocation, resulting in the WWTR1–CAMTA1 fusion, which is considered pathognomonic for EHE. A smaller subset shows YAP1–TFE3 fusions, often associated with distinct histologic features, including more eosinophilic cytoplasm and less myxohyaline stroma [13]. Fluorescence in situ hybridization (FISH) or reverse transcription–polymerase chain reaction (RT-PCR) can confirm these gene fusions and aid in challenging diagnostic scenarios.

### Differential diagnosis

The main differentials for a subcutaneous lesion of this nature include organized hematoma, metastatic carcinoma, epithelioid angiosarcoma, epithelioid sarcoma, and other vascular tumors. Distinguishing EHE from these entities requires integration of morphology, immunohistochemistry, and, wherever feasible, molecular studies.

Organized hematoma is typically excluded by a relevant trauma history, spontaneous resolution, and absence of endothelial immunoreactivity. Metastatic carcinoma is ruled out through the identification of a primary site, cytokeratin positivity, and lack of vascular marker expression. Epithelioid angiosarcoma is differentiated by its higher mitotic activity, marked cytologic atypia, and frequent necrosis. Epithelioid sarcoma generally shows diffuse cytokeratin and EMA positivity along with a granulomatous pattern [14].

### Prognosis and management

Prognostic factors include tumor size >3 cm and mitotic activity >3 mitoses per 50 high-power fields. Deyrup et al. reported that high-risk tumors (meeting one or both of these criteria) had a 59% five-year survival, compared to no disease-related deaths in the low-risk group. Metastases occur in roughly 25% of cases, and the overall five-year mortality is approximately 19% [1].

Surgical excision with tumor-free margins remains the standard of care for localized disease. In high-risk, unresectable, metastatic, or progressive cases, systemic approaches particularly anti-angiogenic agents such as bevacizumab, pazopanib, and sorafenib have shown encouraging results in small series. Multimodal treatment may be indicated when adverse histologic features are present.

## CONCLUSIONS

EHE should be considered in the differential diagnosis of atypical subcutaneous swellings, especially when imaging and cytology are inconclusive. Complete surgical excision with negative margins remains the treatment of choice for localized disease. Given its unpredictable behavior and potential for metastasis, long-term follow-up is essential. Integration of histopathology, immunohistochemistry, and molecular genetics is key to accurate diagnosis and prognostication.

## References

[1] Deyrup AT, Tighiouart M, Montag AG, Weiss SW. Epithelioid hemangioendothelioma of soft tissue: a proposal for risk stratification based on 49 cases. Am J Surg Pathol. 2008; 32:924–27. 10.1097/pas.0b013e31815bf8e6. 18551749

[2] Cirkin DS, Secinti IE, Dogan E, Ozler GS. Epithelioid Hemangioendothelioma in the Tongue: A Rare Case Report. Turk Patoloji Derg. 2023; 39:94–97. 10.5146/tjpath.2021.01560. 34757619 PMC10518129

[3] Stacchiotti S, Miah AB, Frezza AM, Messiou C, Morosi C, Caraceni A, Antonescu CR, Bajpai J, Baldini E, Bauer S, Biagini R, Bielack S, Blay JY, et al. Epithelioid hemangioendothelioma, an ultra-rare cancer: a consensus paper from the community of experts. ESMO Open. 2021; 6:100170. 10.1016/j.esmoop.2021.100170. 34090171 PMC8182432

[4] Folpe AL, Chand EM, Goldblum JR, Weiss SW. Expression of Fli-1, a nuclear transcription factor, distinguishes vascular neoplasms from potential mimics. Am J Surg Pathol. 2001; 25:1061–66. 10.1097/00000478-200108000-00011. 11474291

[5] Rosenberg A, Agulnik M. Epithelioid Hemangioendothelioma: Update on Diagnosis and Treatment. Curr Treat Options Oncol. 2018; 19:19. 10.1007/s11864-018-0536-y. 29546487

[6] Liu YI, Brown SS, Elihu A, Bonham CA, Concepcion W, Longacre TA, Kamaya A. Hepatic epithelioid hemangioendothelioma. Dig Dis Sci. 2011; 56:303–6. 10.1007/s10620-010-1470-4. 21053076

[7] Amin S, Chung H, Jha R. Hepatic epithelioid hemangioendothelioma: MR imaging findings. Abdom Imaging. 2011; 36:407–14. 10.1007/s00261-010-9662-0. 21079951

[8] Sardaro A, Bardoscia L, Petruzzelli MF, Portaluri M. Epithelioid hemangioendothelioma: an overview and update on a rare vascular tumor. Oncol Rev. 2014; 8:259. 10.4081/oncol.2014.259. 25992243 PMC4419652

[9] Zhang Y, Rosenberg AE. Epithelioid Hemangioendothelioma. In: Santini-Araujo E, Kalil RK, Bertoni F, Park YK. (eds) Tumors and Tumor-Like Lesions of Bone. Springer, Cham. 2020. 10.1007/978-3-030-28315-5_38.

[10] Mundada AD, Deodhar K, Ramadwar M, Bal M, Kumar R. Hepatic epithelioid hemangioendothelioma: A clinocopathological correlation. Indian J Pathol Microbiol. 2022; 65:133–36. 10.4103/ijpm.ijpm_350_21. 35074978

[11] Mentzel T, Beham A, Calonje E, Katenkamp D, Fletcher CD. Epithelioid hemangioendothelioma of skin and soft tissues: clinicopathologic and immunohistochemical study of 30 cases. Am J Surg Pathol. 1997; 21:363–74. 10.1097/00000478-199704000-00001. 9130982

[12] Errani C, Zhang L, Sung YS, Hajdu M, Singer S, Maki RG, Healey JH, Antonescu CR. A novel WWTR1-CAMTA1 gene fusion is a consistent abnormality in epithelioid hemangioendothelioma of different anatomic sites. Genes Chromosomes Cancer. 2011; 50:644–53. 10.1002/gcc.20886. 21584898 PMC3264678

[13] Antonescu CR, Le Loarer F, Mosquera JM, Sboner A, Zhang L, Chen CL, Chen HW, Pathan N, Krausz T, Dickson BC, Weinreb I, Rubin MA, Hameed M, Fletcher CD. Novel YAP1-TFE3 fusion defines a distinct subset of epithelioid hemangioendothelioma. Genes Chromosomes Cancer. 2013; 52:775–84. 10.1002/gcc.22073. 23737213 PMC4089994

[14] Choi JH, Ro JY. Epithelioid Cutaneous Mesenchymal Neoplasms: A Practical Diagnostic Approach. Diagnostics (Basel). 2020; 10:233. 10.3390/diagnostics10040233. 32316685 PMC7236000

